# Development and validation of a multiclass LC–MS/MS method for the analysis of cyanotoxins

**DOI:** 10.1007/s00216-025-05829-9

**Published:** 2025-03-27

**Authors:** Lydia Zamlynny, Hannah M. Morris, Sabrina D. Giddings, Johannes Kollatz, Timo H. J. Niedermeyer, Rob C. Jamieson, Daniel G. Beach

**Affiliations:** 1 Department of Civil and Resource Engineering, Dalhousie University, 6299 South St, Halifax, NS Canada; 2 Metrology Research Centre, National Research Council of Canada, 1411 Oxford St, Halifax, NS B3H 3Z1 Canada; 3 Institute of Pharmacy, Freie Universität Berlin, Königin-Luise-Str. 2+4, 14195 Berlin, Germany; 4 Department of Bioorganic Chemistry, Leibniz Institute of Plant Biochemistry, Weinberg 3, 6120 Halle (Saale), Germany

**Keywords:** Cyanobacteria, Microcystins, Cylindrospermopsins, Anatoxins, Aetokthonotoxin, LC–MS/MS, Multiclass analysis, Cyanotoxins

## Abstract

Cyanobacteria are prokaryotic organisms that can form large monospecific blooms, which pose a risk to human and animal health as some species produce toxic secondary metabolites called cyanotoxins. Multiclass cyanotoxin analysis is challenging due to varying chemical and physical properties between classes, as well as potentially large numbers of analogues within each class. Incorporating anatoxins (ATXs) into multiclass methods can be particularly challenging due to their small molecular size, potential interferences, polarity, and a lack of chemical standards for most analogues. Here, we present the development of a multiclass LC–MS/MS method and a quantitative calibration solution for aetokthonotoxin (AETX), an emerging cyanotoxin linked to mass mortalities of bald eagles in the Eastern United States. The developed method is capable of detecting 17 microcystins (MCs), nodularin-R, three cylindrospermopsins (CYNs), AETX, and 17 ATXs, including recently tentatively identified 10-hydroxy analogues. Analytes were identified by retention time and product ion ratio matching with available standards. The method was evaluated with respect to limits of detection (LODs), linear range, accuracy, and precision using neat and matrix matched standards. LODs in wet cyanobacterial biofilms ranged from 0.14 ng/g for CYN to 2.8 ng/g for [Dha^7^]MC-LR with accuracies ranging from 65% for [Leu^1^]MC-LY to 116% for CYN. Finally, the method’s application was demonstrated through analysis of cyanobacterial field samples, a dietary supplement matrix reference material, and passive sampler extracts to assess versatility within different matrices.

**Supplementary Information:**

The online version contains supplementary material available at 10.1007/s00216-025-05829-9.

## Introduction

**Fig. 1:**
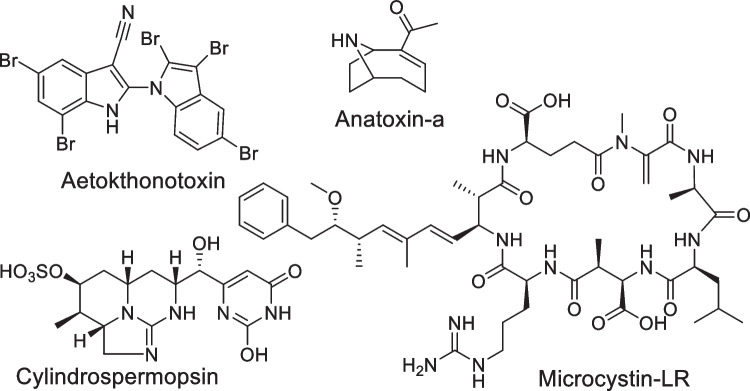
Structures of representative cyanotoxins from structural classes considered in this study

Eutrophication and a changing climate are likely to lead to increased proliferations of cyanobacteria [1–3]. The formation of cyanobacterial blooms is concerning, not only because of the significant ecological impacts that they can have on aquatic environments, but also because some species can produce toxic secondary metabolites known as cyanotoxins. These toxins are split into structural classes, including anatoxins (ATXs), microcystins (MCs), cylindrospermopsins (CYNs), and saxitoxins (STXs), which have been found to cause illness and sometimes death in both humans and animals worldwide (Fig. 1) [1, 4, 5]. Cyanotoxins are produced by a range of globally distributed cyanobacterial taxa and have varying modes of action. More specifically, ATXs and STXs are neurotoxic, where ATX is an agonist of nicotinic acetylcholine receptors and STX inhibits voltage-gated sodium channels [4]. CYNs have multiple routes of toxicity, including hepatotoxicity, while MCs are primarily hepatotoxic [4, 6, 7].

Recently, a new cyanotoxin, aetokthonotoxin (AETX), was identified and implicated in mass mortalities of bald eagles in the Eastern United States (Fig. 1) [8]. This toxin is produced by the epiphytic cyanobacterial species *Aetokthonos hydrillicola*, which grows primarily on aquatic vegetation such as invasive *Hydrilla verticillata*. AETX has been found to cause vacuolar myelinopathy in birds, which leads to a severe loss of motor function and subsequently injury and often death. Although the structure and biosynthetic pathway of this toxin were elucidated, it is not considered in existing cyanotoxin analysis methods, largely due to its novelty and the lack of a chemical standard.

Generally, the risks associated with cyanotoxin production have been attributed to planktonic cyanobacteria, where cells are free floating within the water column, but blooms can also be benthic, where cells proliferate as biofilms attached to bottom substrates, or epiphytic, where cells are attached to other cyanobacteria, algae, or macrophytes [9]. This means that a broad range of sample matrices including water samples, biofilm samples, and extracts from passive sampling devices need to be considered when studying cyanotoxin occurrence.

Many techniques are available to detect and identify cyanotoxins in different matrices including enzyme-linked immunosorbent assays (ELISA), receptor binding assays, protein phosphatase inhibition assays, and liquid chromatography coupled with various detectors [10, 11]. EPA official methods for the detection of MCs and nodularin-R (NOD-R) include determination by ELISA and solid phase extraction with liquid chromatography coupled with mass spectrometry (LC–MS) [12, 13]. Although ELISA methods are relatively simple, they lack information about the toxin profile (i.e., which toxins are present specifically) and a separate assay is needed for each class, which also often require confirmatory analysis using analytical chemistry techniques [14].

LC–MS methods can provide information on toxin profiles and have the potential to detect toxins from multiple classes. However, differences in structures between classes result in varying chemical and physical properties, making the development of multiclass methods challenging. Furthermore, large numbers of analogues within some classes, particularly MCs, need to be considered within these methods. Recently, several new ATXs were discovered in *Microcoleus*-dominated benthic cyanobacterial biofilm samples from Atlantic Canada, and also detected in laboratory strains of planktonic cyanobacteria, but are not yet considered in existing multiclass methods [15]. Despite containing relatively few analogues when compared to MCs, incorporation of ATXs into multiclass methods can be particularly difficult due to their polarity and low molecular weight that can lead to high spectral background at low *m*/*z* in electrospray ionization–MS. Furthermore, interference from phenylalanine (Phe) has led to misidentification of anatoxin-a (ATX) and subsequently false positive results due to similar fragmentation patterns and identical nominal masses [16]. As a result of these challenges, methods have focused on either only detecting ATX or a small number of related compounds without analyzing toxins from other classes.

Recently, there has been a push towards the development of multiclass methods to shorten and simplify cyanotoxin testing protocols, but these methods often still have poor class coverage for ATXs [14]. For example, the EPA official LC–MS method (545) only includes ATX and CYN [17]. Multiclass methods with broader analyte coverage have been developed, but generally focus on incorporating more MC variants rather than improving class coverage or analytical performance of ATXs. Examples of such methods included up to 12 MCs, ATX, and NOD-R [18, 19]. Efforts to expand multiclass analysis further to also include STXs have investigated both reverse phase and hydrophilic interaction liquid chromatography (HILIC) [20, 21]. While HILIC offers better retention and separation of highly polar STXs than reverse phase, it is generally considered to be less robust and shows reduced retention of MCs not containing arginine and a lower resolution separation of ATXs [21, 22].

The aim of this work was to develop a new multiclass LC–MS method that improves selectivity and class coverage of ATXs while also effectively separating and detecting MCs, CYNs, NOD-R, and recently identified AETX in benthic and epiphytic cyanobacterial biofilm samples. Validation parameters investigated included limits of detection, linear range, accuracy, and precision for analytes of each class for which standards were available. The developed method was applied to cyanobacterial samples and passive sampler extracts to confirm its effectiveness in analyzing toxins within different sample types.

## Experimental

### Chemicals and reagents

Certified reference materials (CRMs) for ( +)-anatoxin-a (CRM-ATX), cylindrospermopsin (CRM-CYN), [Dha^7^]-microcystin-LR (CRM-dmMCLR), microcystin-RR (CRM-MCRR), microcystin-LA (CRM-MCLA), microcystin-LR (CRM-MCLR), nodularin-R (CRM-NODR), homoanatoxin-a (CRM-hATX) [23], a pilot-scale freeze-dried cyanobacterial dietary supplement matrix reference material containing multiple classes of cyanotoxins (RM-BGA) [24], an in-house reference material for [Leu^1^]MC-LY, and in-house mixes containing other MCs ([Asp^3^]MC-RR, MC-YR, MC-HtyR, [Asp^3^]MC-LR, MC-LW, MC-LF, MC-HilR, MC-WR, MC-RY, MC-LY) [21] were obtained from the National Research Council of Canada (Halifax, NS, Canada). Standards for dihydroanatoxin-a (H_2_ATX) and ^13^C_4_-( +)-anatoxin-a (^13^C_4_-ATX) were obtained from Gold Standard Diagnostics (Horsham, PA, USA).

Optima LC–MS grade methanol and acetonitrile were obtained from Fisher Scientific (Ottawa, ON, Canada). Sodium borohydride (96% purity) and LC–MS grade formic acid were obtained from Sigma-Aldrich (Oakville, ON, Canada). Deionized water was produced by passing distilled water through a Milli Q Reference A + System (Millipore, Bedford, MA, USA). Tetrahydrofuran-d_8_ (THF-d_8_) was obtained from Cambridge Isotope Laboratories (Tewksbury, MA, USA) and qNMR standard benzoic acid PS1 was obtained from the National Institute of Standards and Technology (Gaithersburg, MD, USA).

### Preparation of AETX calibration solution

AETX was synthesized using a previously reported procedure and its structure and purity assessed by LC–HRMS and NMR spectroscopy [25]. AETX was dissolved in approximately 0.9 g of THF-d_8_ with gentle mixing at room temperature for 24 h. A 0.8 g aliquot was transferred to a 5 mm o.d. precision NMR tube (Wilmad, Vineland, NJ, USA) for quantitation by ^1^H-NMR using a Bruker Avance III spectrometer with an Ultrasheild Plus 700 magnet operating at 16.55 T (Bruker, Fällanden, Switzerland) equipped with a 5-mm cryoprobe at 300 °K using a 90° pulse (value obtained for each tube individually, by calibrating the 360° pulse and dividing by 4). The sweep width was set to 15 ppm and excitation offset was evaluated for each tube separately. A receiver gains of 32 was used for all samples with 4 dummy scans followed by 16 scans and a delay of 40 s. AETX was quantified using a gravimetrically prepared standard of benzoic acid dissolved in THF-d_8_ by external calibration corrected by pulse length [26]. NMR data was processed using MestReNova software version 15.0.1–35.756.

A 0.2 g aliquot of AETX stock solution was quantitatively transferred with degassed methanol into a pre-weighed grade A 200-mL volumetric flask. The solution was brought up to volume using methanol and then mixed for over an hour at ambient temperature using a silicone covered stir bar.

Ampoules (1 mL amber; Duran Wheaton Kimble, Millville, NJ, USA) were pre-rinsed with methanol, dried in an oven overnight, purged with argon, and filled with 500 µL of the prepared AETX calibration solution prior to flame sealing using a Cazzoli FPSI-SS-428 ampouling machine (Cazzoli machine company, Somerset, NJ, USA). Ampoules were individually inspected, labelled in sequential filling order, and stored at − 80 °C. The stability of the calibration solution was assessed by a reverse-isochronous accelerated stability study, where triplicate ampoules were stored at a range of temperature conditions (− 12 °C, 4 °C, 23 °C, and 40 °C) for 2, 7, 14, and 28 days. Samples were analyzed with duplicate injections for each ampoule and compared to results obtained from three control ampoules stored at − 80 °C. The homogeneity of the calibration solution was evaluated by analyzing 12 ampoules from across the fill series and using a single-factor ANOVA to detect heterogeneity.

### Samples and sample extraction

Benthic cyanobacterial biofilms (Fig. S1) were collected from the Wolastoq (Saint John River) near Fredericton (NB, Canada) in August 2019 and from Oat Hill Lake (Dartmouth, NS, Canada) in June 2023. Samples were frozen and thawed to lyse cells before being homogenized and subsampled (approximately 1 g). Subsamples were vortex-mixed with 1 mL of 1:1 (v/v) methanol:water containing 0.1% (v/v) formic acid. Samples were then centrifuged at 21,000 g for 10 min followed by filtration to 0.45 µm using Durapore PVDF membrane centrifugal filters (Millipore, Bedford, MA, USA).

Freeze-dried samples, including RM-BGA [24] and *H. verticillata/A. hydrillicola* [27], were extracted in a similar fashion where approximately 1 mg of *H. verticillata/A. hydrillicola* was extracted with 1 mL of methanol containing 0.1% (v/v) formic acid and 400 mg of RM-BGA was extracted with 40 mL of 1:1 (v/v) methanol:water containing 0.1% (v/v) formic acid. The samples were then vortex-mixed and filtered, as above.

A solid phase adsorption toxin tracking (SPATT) passive sampler [28] was deployed in Indian Brook (Cape Breton, NS, Canada; Fig. S1) for 28 days in the summer of 2022. This sampler contained approximately 2 g of wet activated HP-20 resin (Sigma-Aldrich; Oakville, ON, Canada) and was extracted with methanol using an empty SPE cartridge with a frit (Supelco, Sigma-Aldrich, Oakville, ON, Canada). The extract was brought to dryness using a Savant SPD2010 SpeedVac Concentrator (Thermo Fisher Scientific, Waltham, MA, USA) and then made-up in 1:1 (v/v) methanol:water containing 0.1% (v/v) formic acid. This extract was then centrifuged and filtered as described above.

All samples, with the exception of *H. verticillata/A. hydrillicola*, were spiked with a ^13^C_4_-ATX internal standard (90 µL sample with 10 µL of 600 ng/mL standard) for ATX quantitation.

### Optimized LC–MS/MS method

LC–MS/MS analyses were performed using an Agilent 1290 Infinity liquid chromatography system (Agilent, Santa Clara, CA, USA) coupled to a SCIEX 5500 QTRAP mass spectrometer with a Turbo V ionization source (SCIEX, Concord, Ontario, Canada). An Acquity 1.8 µm HSS T3 column (150 $\times$ 2.1 mm) was fitted with a VanGuard Acquity HSS T3 pre-column (5 $\times$ 2.1 mm; Waters, Milford, MA, USA) and held at 40 °C. Mobile phases A and B were 0.1% (v/v) formic acid in water and acetonitrile, respectively. The first 12 min of the LC method had a flowrate of 0.42 mL/min with a gradient from 2 to 11% B. This was followed by a 10-minute gradient with a flowrate of 0.55 mL/min and ranging from 20 to 100% B. Following this, the mobile phase was kept at 100% B for 4 min (22–26 min) followed by a 4-minute re-equilibration period with 2% B at 0.42 mL/min (total 30 min/sample). An injection volume of 1 µL was used for all samples.

MS/MS data were collected in positive mode for all analytes except AETX for which negative mode was used, using scheduled selected reaction monitoring (SRM) scan mode for all analytes. In positive mode, all analytes used a declustering potential of + 120 V, collision cell exit potential set to + 13 V, and ion spray voltage set to + 3500 V. AETX was analyzed with a declustering potential of − 120 V, collision cell exit potential set to − 25 V, and an ion spray voltage of − 4500 V. Ion source gas flow 1 was set to 45, gas flow 2 to 55 units, and probe temperature to 575 °C for all analytes. Transitions and collision energies (CE) were optimized for each analyte to minimize interferences while maximizing sensitivity. Product ion ratios were determined using responses observed for standards and samples (Table 1). Data analysis was performed in Analyst version 1.6.S2, 2013 (SCIEX).

**Table 1:** Optimized SRM transitions for detected analytes (analyte # corresponds to numbers used in figures and captions to denote analytes)

Analyte #	Analyte	Ret. time (min)	Window (s)	SRM transitions (CE, eV)	Product ion ratio ± std. dev
1	ATX	3.43	60	166.1 > 149.1 (15), 131.1 (25)	1.7 ± 0.2
2	^13^C_4_-ATX	3.43	60	170.0 > 153.0 (15), 135.0 (20)	1.5 ± 0.1
3a	*cis*-H_2_ATX^a^	3.32	120	168.1 > 56.1 (25), 81.1 (20)	3.5 ± 0.5
3b	*trans*-H_2_ATX^a^	4.51			1.1 ± 0.2
4a	10-OH-ATX^b^	3.32	60	168.1 > 150.1 (25), 133.1 (25)	0.53 ± 0.04
4b		5.28			1.6 ± 0.1
5a	10-OH-H_2_ATX^b^	5.67	60	170.1 > 152.1 (25), 124.1 (30)	8.2 ± 0.9
5b		5.95			7.0 ± 0.5
5c		6.07			4.6 ± 0.4
6	hATX	6.66	60	180.1 > 163.1 (20), 145.1 (20)	1.5 ± 0.2
7a	*cis*-H_2_hATX^a^	6.42	60	182.2 > 139.1 (25), 109.1 (30)	10.2 ± 0.5
7b	*trans*-H_2_hATX^a^	8.49		182.2 > 109.1 (30), 139.1 (25)	8.3 ± 0.7
8a	10-OH-hATX^b^	6.06	120	182.2 > 164.1 (20), 105.1 (20)	4.2 ± 0.3
8b		9.55			5.4 ± 0.4
9a	10-OH-H_2_hATX^b^	9.01	120	184.1 > 81.1 (35), 166.1 (20)	2.7 ± 0.1
9b		9.98		184.1 > 166.1 (20), 81.1 (35)	3.82 ± 0.01
9c		10.55			4.46 ± 0.04
10	CYN	2.68	60	416.2 > 194.1 (50), 336.0 (30)	2.1 ± 0.2
11	epi-CYN	2.55	60	416.2 > 194.1 (50), 336.0 (30)	1.6 ± 0.1
12	doCYN	5.57	60	400.2 > 194.1 (45), 320.2 (25)	2.0 ± 0.1
13	MC-LR	15.75	60	995.6 > 213.1 (70), 135.1 (110)	1.8 ± 0.5
14	[Dha^7^]MC-LR	15.82	60	981.5 > 135.1 (120), 121.1 (120)	0.9 ± 0.2
15	[Asp^3^]MC-LR	15.70	60	981.5 > 135.1 (120), 121.1 (120)	14 ± 2
16	MC-RR	14.82	60	519.8 > 135.1 (40). 213.1 (40)	13 ± 3
17	NOD-R	15.36	60	825.5 > 135.1 (95), 227.1 (60)	1.1 ± 0.2
18	MC-YR	15.64	120	1045.6 > 213.1 (75), 135.1 (115)	1.1 ± 0.1
19	MC-RY	16.53	120	1045.6 > 213.1 (75), 135.1 (115)	1.52 ± 0.02
20	MC-HtyR	15.70	60	1059.6 > 213.1 (80), 135.1 (130)	1.7 ± 0.1
21	[Leu^1^]MC-LY	18.81	60	1044.6 > 135.1 (110), 375.2 (50)	1.0 ± 0.3
22	MC-LA	17.92	60	910.5 > 135.1 (90), 375.2 (50)	1.1 ± 0.1
23	MC-LW	18.91	60	1025.5 > 135.1 (105), 375.2 (50)	1.3 ± 0.1
24	MC-LF	19.19	60	986.5 > 135.1 (70), 375.2 (50)	0.94 ± 0.01
25	MC-WR	16.00	60	1068.6 > 135.1 (120), 213.1 (80)	1.8 ± 0.2
26	MC-LY	18.08	60	1002.5 > 135.1 (100), 375.2 (50)	1.7 ± 0.5
27	[Asp^3^]MC-RR	14.69	60	512.8 > 135.1 (40), 329.2 (40)	9.4 ± 0.6
28	MC-HilR	15.92	60	1009.5 > 135.1 (110), 213.1 (70)	0.61 ± 0.06
29	[Leu^1^]MC-LR	16.07	60	519.3 > 135.1 (20), 213.1 (40)	5.1 ± 0.1
30	MC-(H_4_)YR	15.47	60	1049.6 > 213.1 (70), 135.1 (120)	0.75 ± 0.06
31	AETX^c^	21.86	60	649.6 > 489.8 (−70), 410.9 (−80)	2.7 ± 0.1
32	Phe	3.95	60	166.1 > 120.1 (15), 149.1 (15)	32 ± 2

^a^*cis* and *trans* notation as introduced by Mann et al. (2012) [29]

^b^Epimer configurations not defined

^c^Acquired in negative mode

### Validation

Method validation was carried out by preparing two calibration curves with a concentration range spanning 0.014–270 ng/mL for nine analytes with available reference materials (CYN, MC-RR, [Dha^7^]MC-LR, MC-LR, MC-LA, [Leu^1^]MC-LY, NOD-R, ATX, and hATX) as well as AETX, for which a new quantitative calibration solution has been prepared as part of the current study. One curve was prepared neat in 1:1 (v/v) methanol:water with 0.1% (v/v) formic acid and the other was prepared in an extract of non-toxic *Microcoleus*-dominated benthic cyanobacterial biofilm collected from the Wolastoq near Fredericton (NB, Canada) to evaluate the impacts of this matrix on analysis. Limits of detection (LODs) were evaluated by calculating the concentration at which the signal to noise ratio (S/N) was equal to three using the two lowest detected standards analyzed in triplicate for all analytes except AETX where the LOD was found by multiplying the signal observed in blank solvent injections by three since some low-level carryover was observed for this analyte. The linear range for each analyte was defined as the range where the concentration dependence of analyte response (defined as peak area/concentration) was within two standard deviations of the mean concentration dependence of analyte response [30]. Recovery and precision of the extraction procedure and method were evaluated by gravimetrically spiking the same non-toxic *Microcoleus*-dominated benthic cyanobacterial biofilm as above, which had previously been frozen and thawed to lyse cells, to a final concentration of 20 ng/mL for each analyte. For evaluation of AETX recoveries, a non-toxic extract of *H. verticillata*/*A. hydrillicola* was used to simulate the correct matrix. The spiked mixture was vortex mixed and then extracted following the extraction procedure outlined above (1 g spiked biofilm and 1 mg spiked *H. verticillata/A. hydrillicola*, respectively, extracted with 1 mL of solvent) in triplicate.

ATX was calibrated by double-isotope dilution using a linear calibration model, with peak area ratio of ATX:^13^C_4_-ATX measured in place of peak area in samples and calibration curves. All other analytes were externally calibrated using the neat solvent calibration curve and a linear calibration model.

## Results and discussion

### AETX calibration solution

A calibration solution for AETX was prepared at 1.07 ± 0.03 µg/mL in methanol from synthetic AETX. Value assignment was carried out by quantitative ^1^H-NMR of a concentrated stock solution in THF-d_8_ using external calibration with a benzoic acid CRM. An accelerated stability study did not detect any degradation up to the highest temperatures investigated (40 °C) over the course of 28 days (Fig. S2), and no inhomogeneity was observed across the fill series. This calibration solution will be useful for standardizing AETX measurements across time and between laboratories in ongoing investigations into its occurrence, toxicity and impacts. These experiments demonstrate that production of a CRM for AETX would be highly feasible, should a broader research or regulatory need be established in the future.

### LC–MS method development

**Fig. 2:**
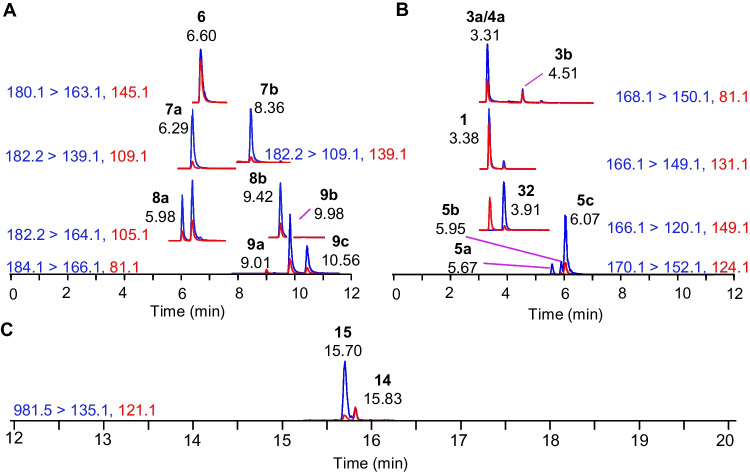
Extracted ion chromatograms of **A** hATX (**6**), *cis*-H_2_hATX (**7a**), *trans*-H_2_hATX (**7b**), 10-OH-hATX (**8a**, **8b**), and 10-OH-H_2_hATX (**9a**–**c**) from a cyanobacterial biofilm collected in NS and **B** ATX (**1**), *cis*-H_2_ATX (**3a**), *trans*-H_2_ATX (**3b**), 10-OH-ATX (**4a**), 10-OH-H_2_ATX (**5a**–**c**), and Phe (**32**) from a benthic cyanobacterial biofilm collected in NB, and **C** [Dha^7^]MC-LR (**14**) and [Asp^3^]MC-LR (**15**) detected in RM-BGA with quantifier transitions shown in blue and qualifier transitions in red

A reverse phase separation was developed by combining a gradient previously developed for ATXs with another developed for the analysis of MCs [15, 31]. This combined gradient was expanded further to include AETX and was also found to be effective for analysis of NOD-R, and CYNs. STXs were not sufficiently retained in reverse phase LC to be reliably analyzed by the current method. The flowrate was increased to decrease method run time while maintaining separations between key analyte pairs that are challenging to resolve by mass spectrometry alone, including ATX and Phe, 10-OH-hATX, and *cis*-H_2_hATX as well as [Dha^7^]MC-LR and [Asp^3^]MC-LR (Fig. S3, Fig. 2). Additionally, all other recently tentatively identified 10-OH-ATXs were incorporated, significantly improving class coverage of ATXs when compared to other methods (Fig. 2) [17–20]. In the final method, ATXs and CYNs eluted between 2.5 and 12 min, MCs between 12 and 20 min, and AETX near 22 min (Fig. S4). A relatively low injection volume of 1 µL was used for all analyses, which was required to maintain good peak shape and resolution of the polar anatoxins in 1:1 (v/v) methanol:water extracts.

**Fig. 3:**
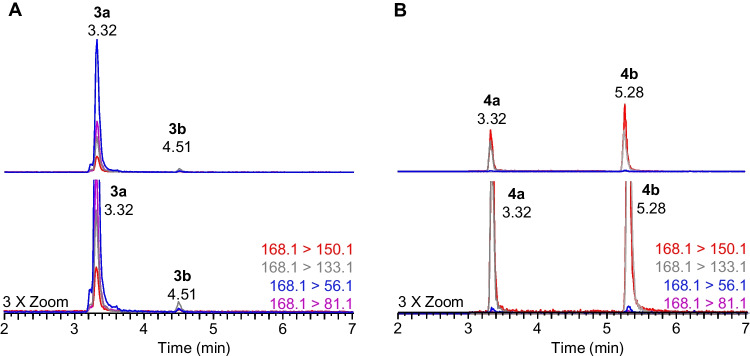
**A** Extracted ion chromatograms of a standard containing isomers of H_2_ATX (**3a**,** 3b**) and **B** a semisynthetic preparation of 10-OH-ATX (**4a**, **4b**) [15], where the lower panels show a 3 × magnified version of the top panels showing interference in retention time and some SRM transitions

Selective scheduled SRM transitions were chosen based on either previous studies, infusion experiments with available standards, or product ion scans of RM-BGA and field samples for analytes that did not have available standards [15, 21]. Particular focus was dedicated to resolving *cis*-H_2_ATX and 10-OH-ATX, since this isomeric pair was not effectively resolved chromatographically. However, since these analytes have similar fragmentation patterns (Fig. S5), fully selective transitions for either analyte were not identified. The H_2_ATX transitions (168.1 > 56.1 and 81.1) were found to be partially selective as their responses were comparatively low for samples containing only 10-OH-ATXs unlike the 10-OH-ATX transitions (168.1 > 150.1 and 133.1), which had strong signals for samples containing only H_2_ATXs (Fig. 3). Since H_2_ATX can be detected more selectively, once standards become available for both H_2_ATX and 10-OH-ATX, it would be possible to quantify 10-OH-ATX by subtracting the measured concentration of H_2_ATX from the measured concentration of 10-OH-ATX.

Overall, the optimization of both the gradient and detection parameters yielded a multiclass method with significantly improved class coverage for ATXs, capable of separating and detecting 17 ATXs, 17 MCs, NOD-R, three CYNs, and AETX. This could be expanded in the future to include additional MC and ATX analogues as standards become available.

### Validation

Method performance was evaluated with respect to LODs, linear range, recovery, precision, and matrix effects for analytes for which certified reference materials were available (CYN, MC-RR, [Dha^7^]MC-LR, MC-LR, MC-LA, [Leu^1^]MC-LY, NOD-R, ATX, and hATX), as well as for AETX, for which a quantitative calibration standard was prepared.

**Fig. 4:**
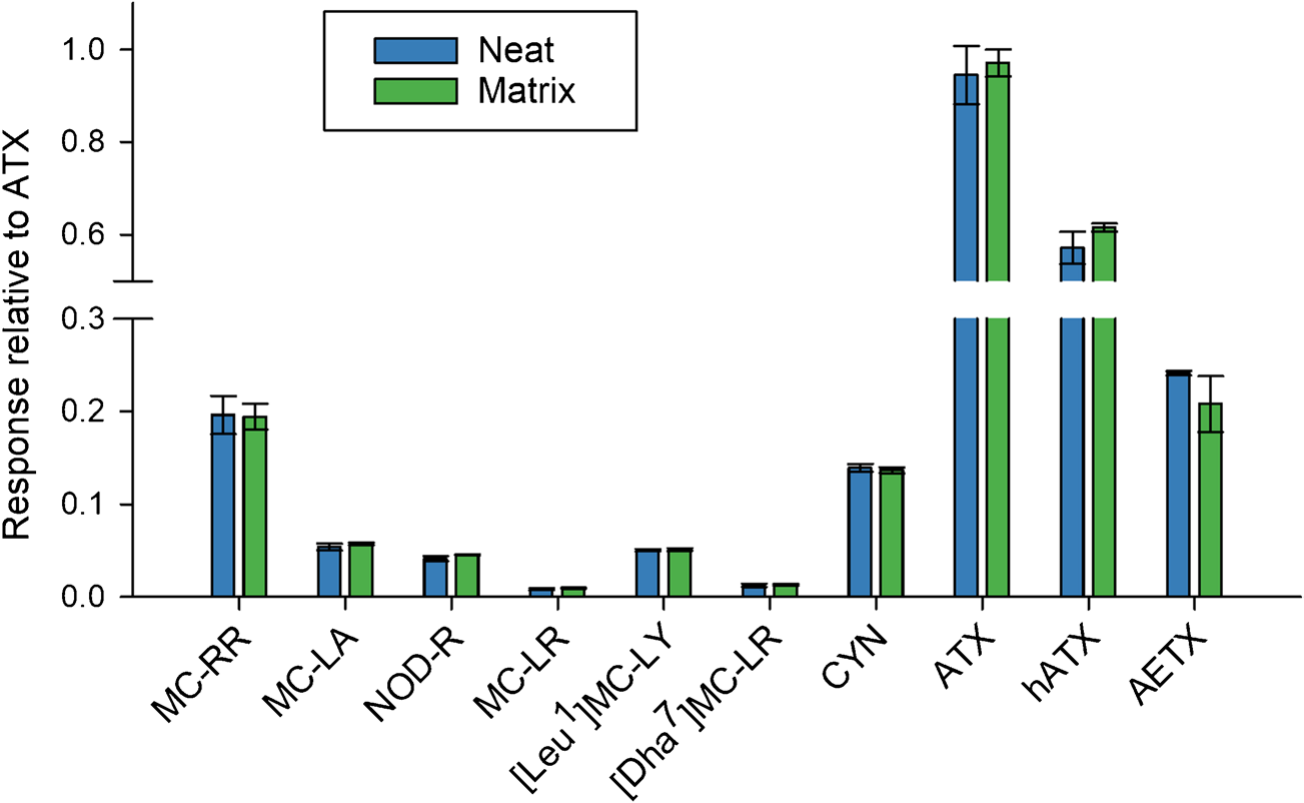
Comparison of calibration slopes relative to ATX calculated from a calibration curve prepared in 1:1 (v/v) methanol:water with 0.1% (v/v) formic acid with a calibration curve prepared in a non-toxic cyanobacterial biofilm extract with 0.1% (v/v) formic acid, where error bars represent the standard deviations between replicate curves

Matrix effects for available benthic biofilm samples were investigated by comparing the responses of two calibration curves, one prepared in neat extraction solvent and the other prepared in a non-toxic cyanobacterial biofilm extract. Triplicate injections of both curves demonstrated that matrix effects were minimal for all analytes investigated as responses were not found to vary significantly between curves (RSDs within 10% with the exception of AETX with an RSD of 12%; Fig. 4). Therefore, external calibration was found to be acceptable for analysis of all analytes in the benthic biofilms considered, but further evaluation of other matrices is recommended. Finally, to aid in the identification of ATX, prevent matrix interference and associated biases, double-isotope dilution was implemented for ATX as a secondary confirmatory measure.

The linear range was investigated for each analyte over a range spanning 0.014–270 ng/mL and in all cases the maximum value was limited by the concentration of the prepared standards (270 ng/mL) and not non-linearity of the method (Table 2, Figs. S6 and S7).

**Table 2:** Figures of merit determined from validation experiments for the developed method

Analyte	Linear range^a^		*R*^2^	LOD^b^ (ng/g)	Recovery (%)	Precision^d^ (% RSD)
	Min (ng/mL)	Max (ng/mL)				
ATX	1.1	270	0.9999	0.18	108	2.3
hATX	0.37	270	0.9999	0.35	103	2.3
CYN	0.37	270	0.9999	0.14	116	0.84
MC-RR	0.37	270	0.9994	0.18	105	2.5
[Dha^7^]MC-LR	3.3	270	0.9993	2.8	88	0.90
MC-LR	1.1	270	0.9995	1.8	94	9.2
MC-LA	1.1	270	0.9999	0.67	87	1.5
[Leu^1^]MC-LY	1.1	270	0.9993	1.2	65	6.7
NOD-R	1.1	270	0.9999	0.72	109	0.76
AETX	0.12	270	0.9988	0.11^c^	78	5.3

^a^Range investigated: 0.014–270 ng/mL for all analytes

^b^LOD is defined as the concentration at which S/N = 3 was observed/predicted

^c^LOD is determined by multiplying the concentration observed in blank solvent injections by 3

^d^Precision is defined as the relative standard deviation of results between triplicate extraction and quantitation of toxins in recovery experiments

Although guidelines for some cyanotoxins have been developed for drinking and recreational water by the World Health Organization (WHO) and Health Canada, there are currently no regulatory values for cyanotoxins in benthic biofilms [1, 32, 33]. However, the LODs determined in this study (Table 2) can be compared to existing water guidelines to evaluate the method’s suitability for direct analysis of cyanotoxins in water. In this case, ng/g units are equivalent to µg/L, which takes into account the 1:1 dilution with methanol in the current protocol. The developed method had sufficiently low LODs for effective analysis of all analytes below recreational guidelines provided by the WHO [1]. LODs for ATXs and CYNs also fell below WHO drinking water guidelines, but larger injection volumes or additional preconcentration would be required to meet WHO guidelines and Health Canada regulations for MCs in drinking water [1, 32].

Method precision and extraction recoveries were evaluated by using a mixture of CRMs to spike a non-toxic benthic cyanobacterial biofilm sample and following typical extraction procedures. ATXs and CYN were found to have higher recoveries and better precision than MCs (Table 2), but overall method performance was found to be in good agreement with values reported for other multiclass LC–MS methods [18, 34, 35]. The recovery of AETX was evaluated by spiking the prepared AETX standard into samples of freeze-dried *H. verticillata*/*A. hydrillicola* that did not contain detectable levels of the toxin to simulate the correct sample matrix. The recovery of this novel toxin fell within the range observed for other cyanotoxin classes and the method was therefore found to be suitable for its analysis.

### Application to cyanobacterial samples

**Fig. 5:**
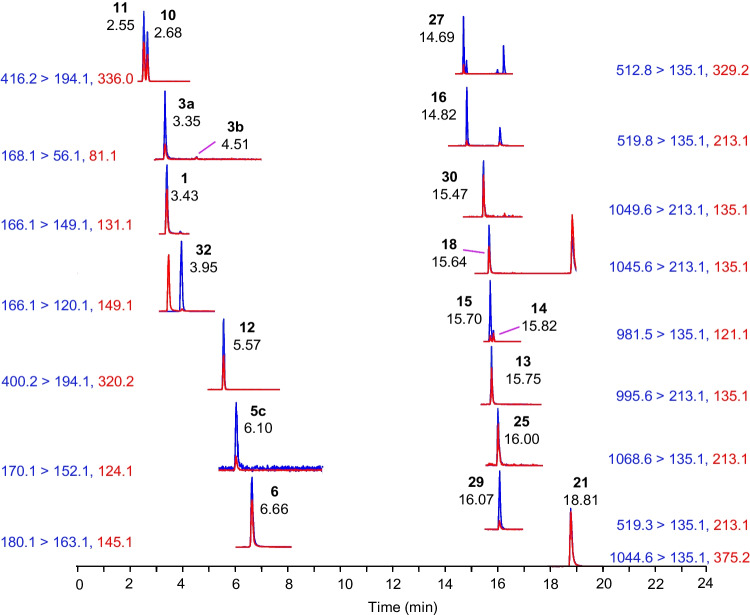
Extracted ion chromatograms of analytes detected in RM-BGA including ATX (**1**), H_2_ATXs (**3a**, **3b**), 10-OH-H_2_ATX (**5c**), hATX (**6**), CYN (**10**), epi-CYN (**11**), doCYN (**12**), [Asp^3^]MC-RR (**27**), MC-RR (**16**), MC-(H_4_)YR (**30**), MC-YR (**18**), MC-LR (**13**), [Asp^3^]MC-LR (**15**), [Dha^7^]MC-LR (**14**), MC-WR (**25**), [Leu^1^]MC-LR (**29**), and [Leu^1^]MC-LY (**21**) with quantifier transitions in blue and qualifier transitions in red. Phe (**32**) was monitored as a common interference to confirm resolution from ATX

The broad applicability of the developed method was demonstrated through analysis of a dietary supplement matrix reference material (RM-BGA), benthic cyanobacterial biofilms, and passive sampler extracts [24]. This was done to investigate method versatility during analysis of cyanotoxins within different matrices to evaluate the impacts of any potential interferences and possible expansion for analysis of other sample types. The analysis of RM-BGA confirmed effective method performance and retention time alignment for all contained analytes from each class and matched expected results obtained from calibration standards (Fig. S4 and Fig. 5).

Good performance and robustness were observed for analysis of ATXs in benthic cyanobacterial biofilms, which were analyzed across large sample sets (> 200 samples) as part of an ongoing study into occurrence and drivers of *Microcoleus* in Nova Scotia, Canada (result to be reported elsewhere). Concentrations for validated ATXs in benthic biofilms were compared to results obtained using a previously reported class-specific LC–HRMS method [15] and found to be in good agreement with a maximum variation of 11% (Table 3).

**Table 3:** Comparison of results obtained for validated ATXs (ATX and hATX) to results obtained from a reference method [15] in benthic biofilms collected from the Wolastoq (near Fredericton, NB, Canada) and Oat Hill Lake (Dartmouth, NS, Canada)

	Biofilm from NB			Biofilm from NS		
Analyte	LC–HRMS^a^ (µg/g)	LC–MS/MS (µg/g)	Difference (%)	LC–HRMS^a^ (µg/g)	LC–MS/MS (µg/g)	Difference (%)
ATX	11.7	11.4	2.1	0.219	0.243	11.1
hATX	0.118	0.120	1.8	10.2	9.88	3.2

^a^LC–HRMS reference measurements were made using a previously described class-specific method for anatoxins [15]

**Fig. 6:**
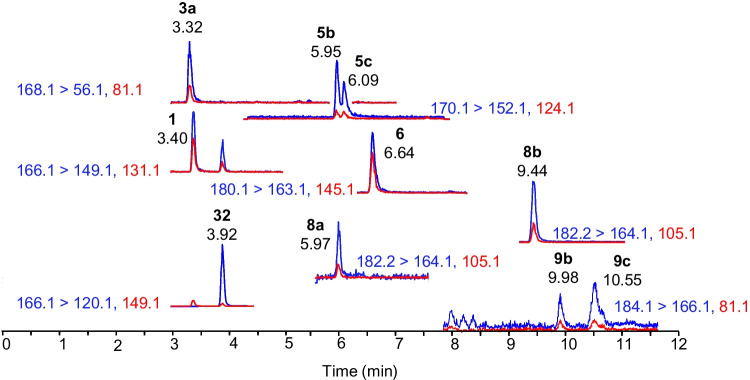
Extracted ion chromatograms of ATX (**1**), *cis*-H_2_ATX (**3a**) 10-OH-H_2_ATX (**5b**, **5c**), hATX (**6**), 10-OH-hATX (**8a**, **8b**), 10-OH-H_2_hATX (**9b**, **9c**), and Phe (**32**) from a passive sampler deployed in Indian Brook (Cape Breton, NS, Canada) where quantifier transitions are shown in blue and qualifier transitions in red

The use of passive sampling, particularly using the SPATT approach, has become popular in the field of cyanotoxin analysis due to its ability to provide a time-averaged measure of cyanotoxin presence in a waterbody and to mitigate the very high spatial and temporal variability in grab sampling [28]. However, extracts from passive samplers represent an extremely complex chemical matrix and can yield false positive results with some analysis methods, particularly for ATXs. An example of a passive sampler extract showing low levels of ATXs analyzed using the current method showed good retention time alignment for all analytes detected and an absence of chemical interference (Fig. 6), despite this matrix being generally more complex and having shown higher background and potential for low-level false positive detections with previous methods.

**Fig. 7:**
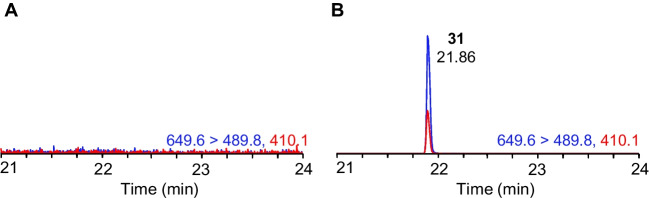
Extracted ion chromatograms of AETX (**31**) as detected in **A** a negative extract of *H. verticillata/A. hydrillicola* (sample #58 in Table 4) and **B** a positive extract of *H. verticillata/A. hydrillicola* (sample #17 in Table 4) with quantifier transitions shown in blue and qualifier transitions in red

Finally, extracts of *H. verticillata/A. hydrillicola* were analyzed to confirm detection of AETX in environmental samples (Fig. 7). The obtained results demonstrated good performance and selectivity without an impact on retention time or product ion ratio as compared with the prepared AETX calibration solution (Fig. S4). Quantitative results generated using the developed method and calibration solution were in good agreement with previous results of peak area-based detection from LC–MS and the presence or absence of genes associated with AETX biosynthesis measured by PCR (Table 4) [27]. The developed method and standard will help generate quantitative occurrence data for this toxin beyond the original study sites in the Eastern United States, which will provide a more accurate representation of the associated risks [8]. It will also help standardize measurements in ongoing research into the occurrence and broader ecological and potential health impacts of AETX.

**Table 4:** Concentration of AETX identified in extracts of *H. verticillata/A. hydrillicola* compared to previously reported results [27]

Sample ID^a^	AETX concentration (ng/mg)	Peak area^a^ (mg^−1^)	PCR result^a^
9	17.5	9.07 $\times$ 10^5^	+
17	700	1.59 $\times$ 10^8^	+
28	225	5.70 $\times$ 10^7^	+
50	0	−	−
58	0	−	−

^a^As reported by Štenclová et al. 2023 [27]. For PCR, “ + ” indicates a positive result for *aetA*, *aetB*, *aetC* genes and ITS1, ITS2 domains associated with AETX biosynthesis in *Aetokthonos hydrillicola*, while “−” indicates a negative result for these genes

## Conclusions

A multiclass method suitable for simultaneous analysis of 17 ATXs, three CYNs, 17 MCs, NOD-R, and AETX was developed. Method performance was evaluated with respect to LODs, precision, and accuracy for all analytes for which reference materials were available. A quantitative calibration standard for AETX was developed to allow for accurate quantitation of this novel toxin. External calibration was suitable for all analytes investigated as matrix effects in benthic biofilms were found to be minimal. Furthermore, the extraction procedure yielded acceptable recoveries of analytes and LODs suitable for analysis of all toxins below recreational water guidelines provided by the WHO. The developed method was applied to a variety of benthic cyanobacterial biofilm samples, passive samplers, and matrix reference materials to demonstrate its performance within different matrices. Finally, the method was found to effectively separate and detect the newly identified ATXs and AETX and should therefore be a useful tool for research and routine monitoring of cyanotoxins from different structural classes.

## Supplementary Information

Below is the link to the electronic supplementary material.

ESM 1 Supplementary file1 (PDF 430 KB)
