# Supplementary material for: Development and validation of a multiclass LC–MS/MS method for the analysis of cyanotoxins
Source: Anal Bioanal Chem. 2025 Mar 27;418(2):667–78. doi: 10.1007/s00216-025-05829-9 (PMC12783199; doi:10.1007/s00216-025-05829-9)
Supplement: Supplementary file 1 — Supplementary file1 (PDF 430 KB) [file 216_2025_5829_MOESM1_ESM.pdf]

Supplementary Information

Development and Validation of a Multi-Class LC–MS/MS Method for the Analysis of Cyanotoxins

Lydia Zamlynny[1,2], Hannah Morris[1], Sabrina D. Giddings[2], Johannes Kollatz[3,4], Timo H. J. Niedermeyer[3], Rob C. Jamieson[1], Daniel G. Beach[1,2*]

[1]Dalhousie University, Department of Civil and Resource Engineering, 6299 South St., Halifax, NS, Canada
[2]Metrology Research Centre, National Research Council of Canada, 1411 Oxford St., Halifax, NS, Canada
[3]Institute of Pharmacy, Freie Universität Berlin, Königin-Luise-Str. 2+4, 14195 Berlin, Germany
[4]Department of Bioorganic Chemistry, Leibniz Institute of Plant Biochemistry, Weinberg 3, 6120 Halle (Saale), Germany

*Corresponding author:
Tel.: +1 (902) 426-8274
E-mail address: daniel.beach@nrc-cnrc.gc.ca
Dr. Daniel G. Beach
Biotoxin Metrology
National Research Council of Canada
1411 Oxford Street

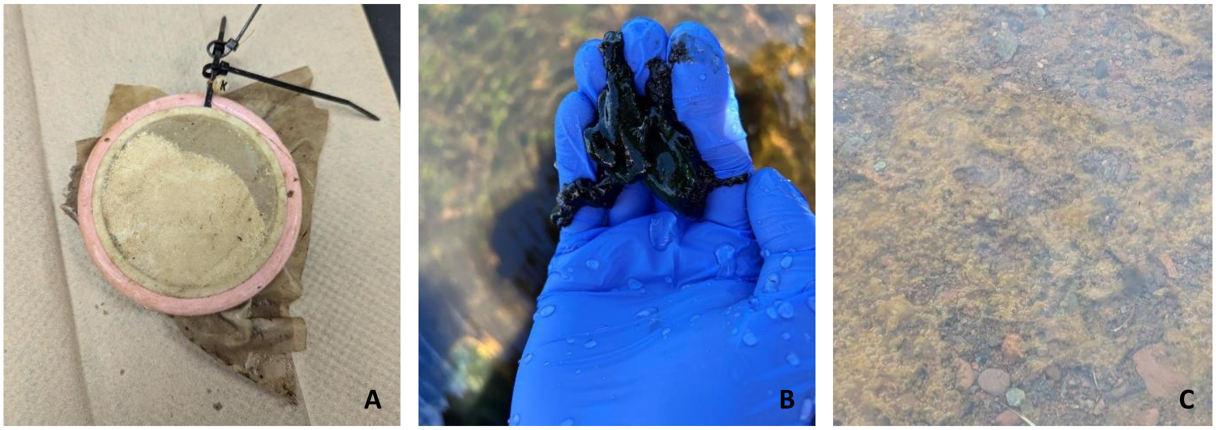

Fig. S1 **A**: passive sampler extract containing activated HP-20 resin. **B**: collected biofilm sample and **C**: benthic cyanobacterial mats/biofilm observed in the French River (Tatamagouche, NS, Canada)

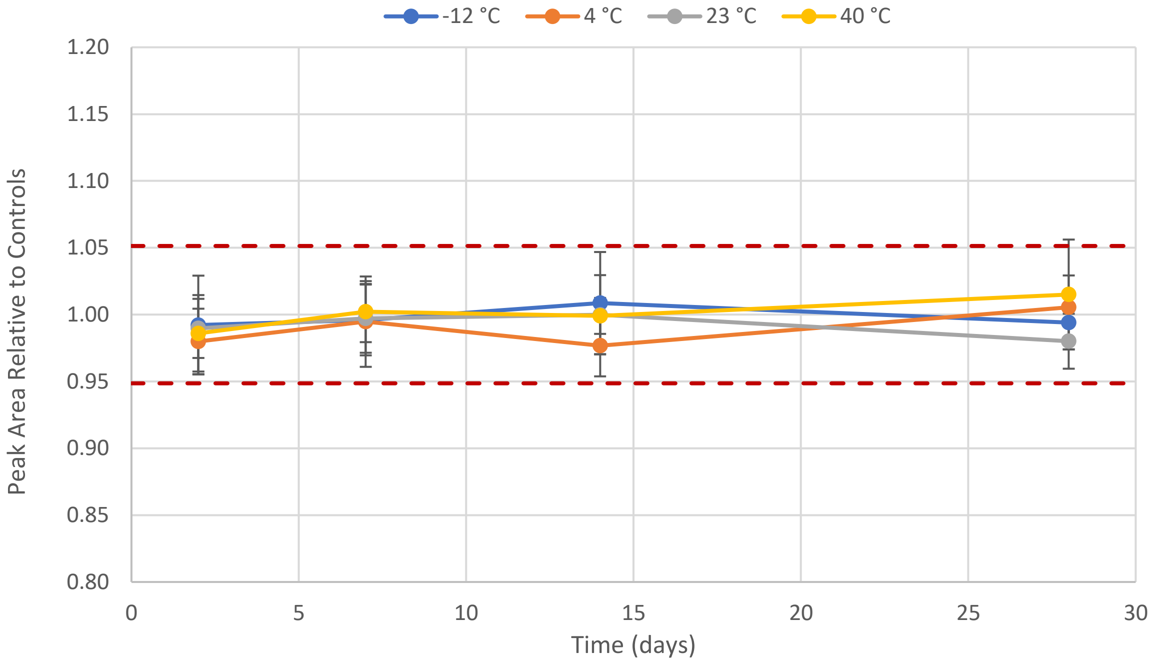

Fig. S2 Stability of the prepared AETX calibration solution in argon-purged flame-sealed ampoules over 28 days at a range of storage temperatures (-20 °C, 4 °C, 23 °C, and 40 °C) where error bars are standard deviations between replicate ampoules at each testing condition and the dashed red line represents the range of responses within two standard deviations of the control average (average of analyzed samples stored at -80 °C)

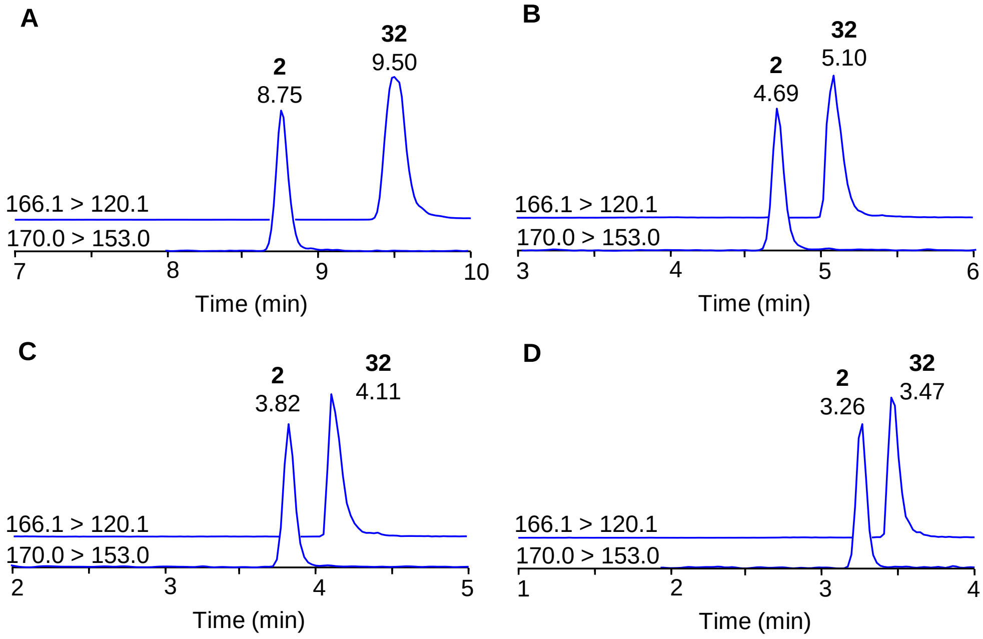

Fig. S3 Extracted ion chromatograms of $^{13}C_4$-ATX (**2**) and Phe (**32**) corresponding to gradients ranging from 2 – 11 % of mobile phase B with **A**: a flowrate of 0.2 mL/min over 20 minutes, **B**: a flowrate of 0.3 mL/min over 15 minutes, **C**: a flowrate of 0.4 mL/min for 12 minutes, and **D**: a flowrate of 0.5 mL/min over 10 minutes. The resolution between peaks in each panel was determined to be 3.7, 2.3, 1.9, and 1.4 for panels **A** – **D,** respectively

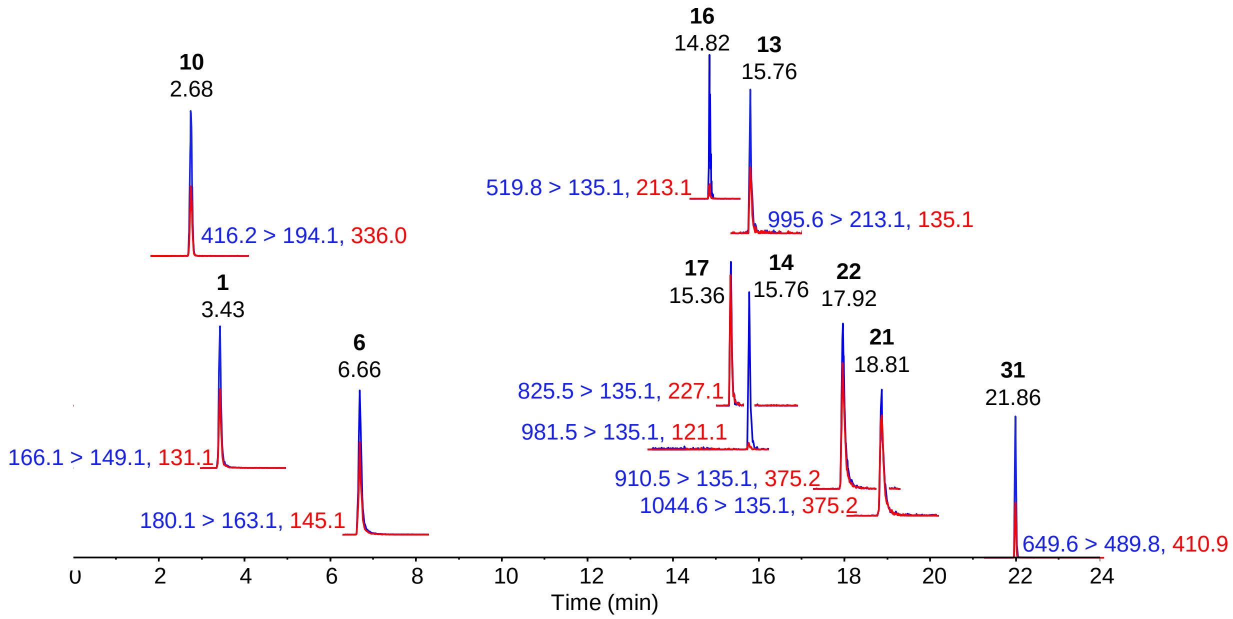

Fig S4 Extracted ion chromatograms of CRM solutions for ATX (**1**), CYN (**10**), hATX (**6**), MC-RR (**16**), MC-LR (**13**), NOD-R (**17**), [Dha[7]]MC-LR (**14**), and MC-LA (**22**), [Leu[1]]MC-LY (**21**), as well as the reference material developed for AETX (**31**), where quantitative transitions are shown in blue and qualitative transitions are red

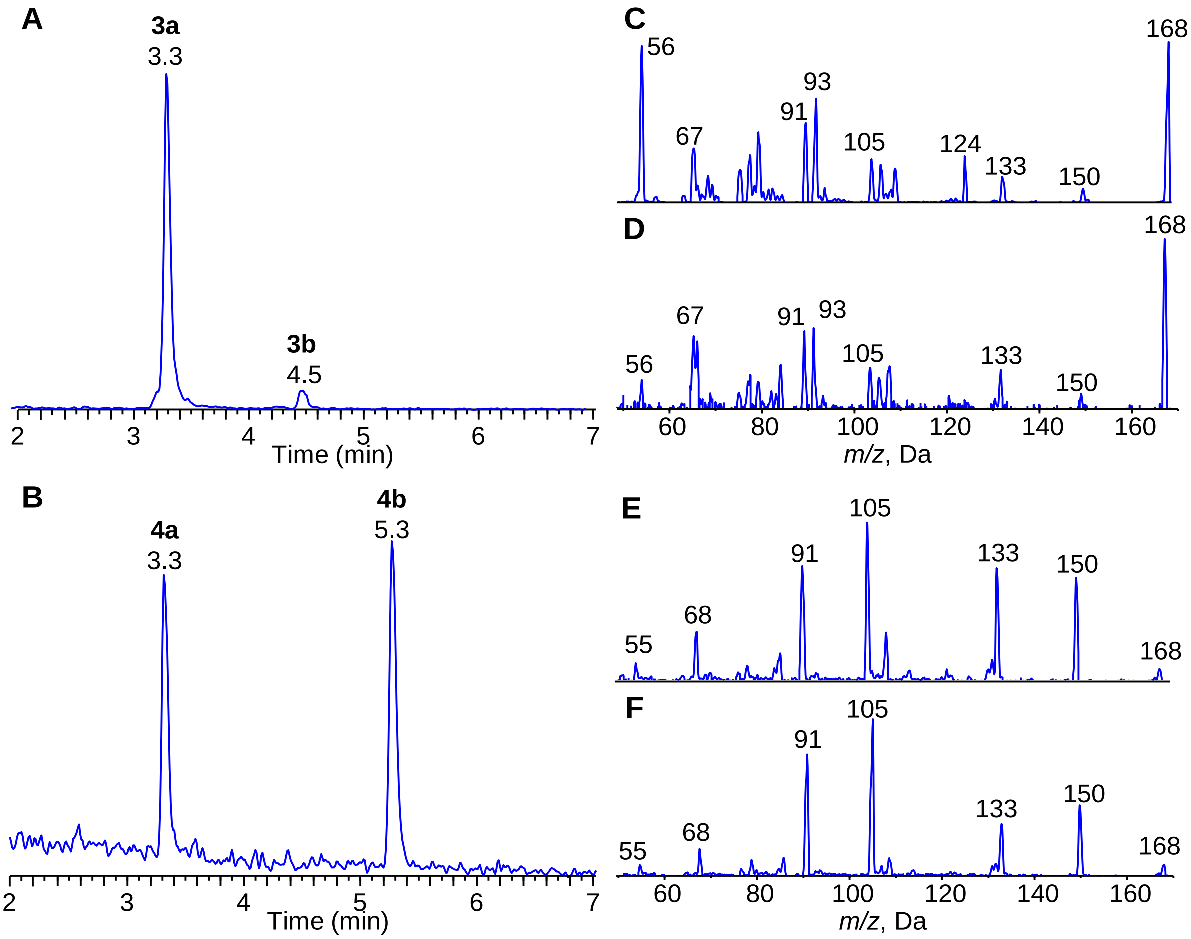

Fig. S5 LC–MS/MS analysis of isomers of *cis*-H_2ATX (**3a**) and *trans*-H_2ATX (**3b**) in a commercial standard (**A, C, D)** and 10-OH-ATX (**4a** and **4b**) formed through the reaction between ATX and $NaBH_4$ (**B, E, F**) showing the fragmentation spectra of each chromatographic peak where **C** and **D** were obtained from the peaks in **A** at 3.3 and 4.5 minutes respectively and **E** and **F** were obtained from peaks in **B** at 3.3 and 5.3 minutes, respectively

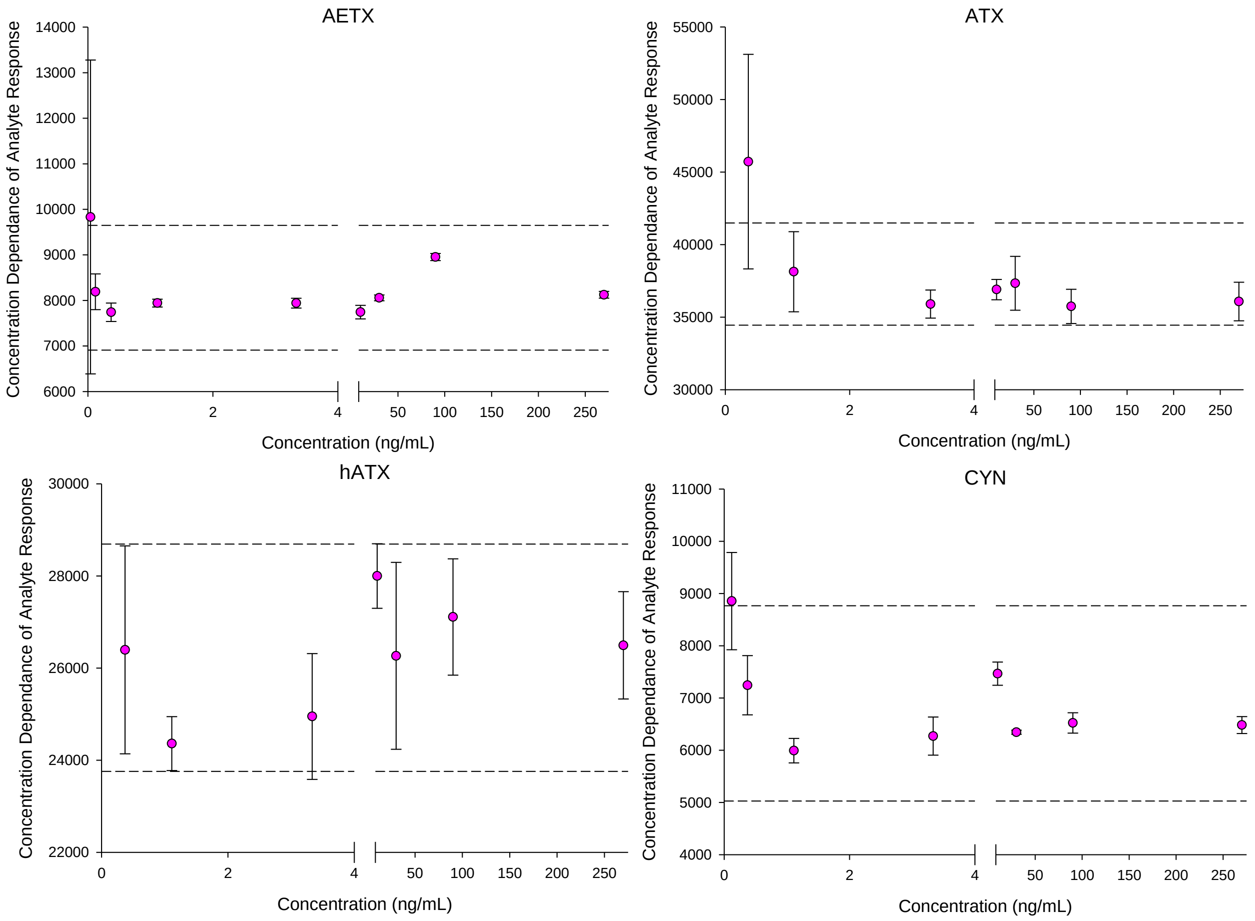

Fig. S6 Concentration dependance of analyte response (peak area/concentration) for ATXs, AETX, and CYN where error bars represent the standard deviation of replicate analysis at each concentration. Linear range for each analyte is the range of concentrations that fall within two standard deviations of the mean concentration dependance of analyte response for all detected levels of each analyte (dashed lines)

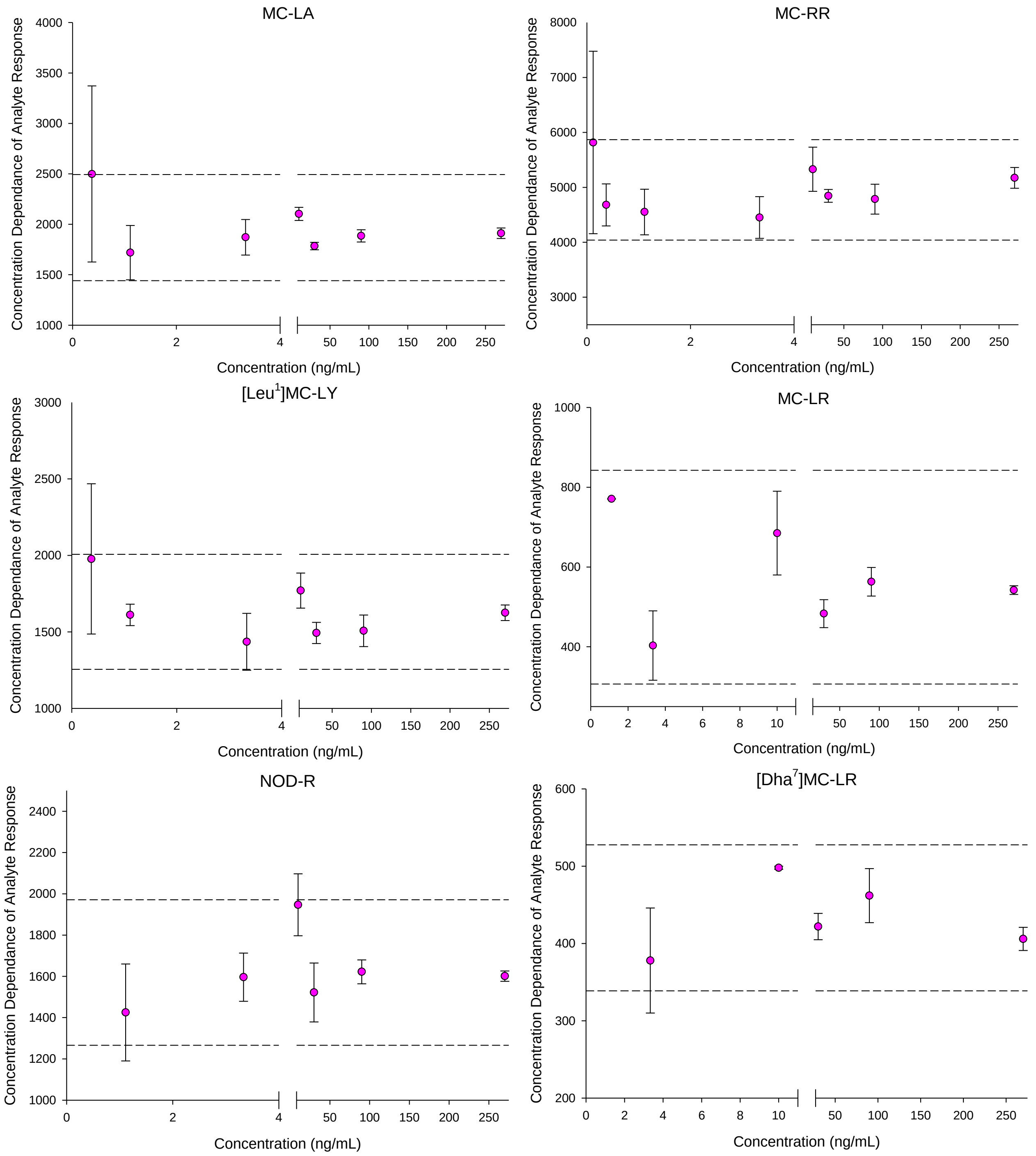

Fig. S7 Concentration dependance of analyte response (peak area/concentration) for MCs and NOD-R where error bars represent the standard deviation of replicate analysis at each concentration. Linear range for each analyte is the range of concentrations that fall within two standard deviations of the mean concentration dependance of analyte response for all detected levels of each analyte (dashed lines)
